# Treatment of Type 2 diabetes with Tianqi Jiangtang Capsule: A systematic review and meta-analysis of randomized controlled trials

**DOI:** 10.1097/MD.0000000000019702

**Published:** 2020-05-22

**Authors:** Chunli Piao, Qi Zhang, De Jin, Mengsu Shao, Chaoran Bi, Li Wang, Cheng Tang, Fengmei Lian, Xiaolin Tong

**Affiliations:** a Institution of Shenzhen Hospital, Guangzhou University of Chinese Medicine (Futian), Shenzhen, Guangdong; b Changchun University of Chinese Medicine, Changchun, Jilin; c Institution of Guang’anmen Hospital, China Academy of Chinese Medical Science, Beijin, China.

**Keywords:** add-on therapy, diabetes, meta-analysis, randomized controlled trials, Tianqi Jiangtang capsule

## Abstract

**Background:**

Tianqi Jiangtang Capsule is a commonly used Chinese patent medicine for the treatment of type 2 diabetes mellitus (T2DM) in China. The purpose of this study is to systematically evaluate the clinical efficacy and safety of Tianqi Jiangtang Capsule in the treatment of type 2 diabetes mellitus.

**Methods:**

Randomized controlled trials (RCTs) of Tianqi Jiangtang Capsule in the treatment of type 2 diabetes mellitus were retrieved. According to the requirements of Cochrane Manual, the included literature was assessed and meta-analyzed with RevMan 5.3 software.

**Results:**

(1) Meta-analysis included 8 RCTs and 1029 participants.

(2) There were two studies on adverse reactions.

(3) Meta-analysis showed that Tianqi Jiangtang Capsule could significantly reduce HbA1c (n = 1029; MD, −0.31; 95% CI, [−0.43 to −0.19]; *P* < .00001; I^2^ = 0%). FBG (Z = 4.28 (*P* < .0001), MD = 0.78, 95%CI[−1.14 to −0.43]). 2hPG [OR = −1.25, 95% CI [−1.25 to −0.65], Z = 6.26 (*P* < .00001)] compared with the control group.

**Conclusions:**

According to the results of this study, Tianqi Jiangtang Capsule combined with antidiabetic agents may have a better therapeutic effect on diabetes mellitus than antidiabetic agents alone, but due to the low methodological quality and limited number of studies, more high-quality studies are needed to verify it.

## 1 Introduction

With the development of social economy and the increasing pressure of people's life and work, diabetes has changed from a rare disease to an epidemic. In 2015, an estimated 415 million adults suffered from diabetes, accounting for 9.1% of the global adult population. China has the largest number of diabetic patients in the world, up to 109 million.^[1]^ Monitoring of chronic diseases and risk factors for these diseases in China in 2013 showed that the prevalence of diabetes in people aged 18 and over was 10.4%.^[2]^ As we all know, Diabetes mellitus is a complex, chronic illness, and 60% to 70% of those with diabetes also have obesity and 70% to 80% have hyperlipidemia.^[3,4]^ In addition, diabetes mellitus comorbidities and medical requirements contribute to high and rising healthcare costs.^[5]^ According to the American Diabetes Association's recommendation for the treatment of diabetes, metformin is a first-line drug and is widely used clinically.^[6]^ Since type 2 diabetes is a progressive and multifactorial condition, patients invariably require additional antihyperglycemic therapy.^[7]^ When metformin is taboo or intolerant and inefficient, drug combination therapy is widely used in clinical practice. Despite the benefits the combination, it also increases the risk of adverse events. In the clinical use of metformin and other hypoglycemic drugs, it might lead to some adverse events, such as edema, lactic acidosis, hypoglycemia, etc.^[8]^ Therefore, many physicians are trying to find new strategies for drug combination at present. Due to the side effects and drug resistance of drugs, more and more clinicians in our country are accelerating their expansion to the field of traditional drug treatment in the practice of diabetes treatment.

Traditional Chinese medicine (TCM) as a treatment of diabetes mellitus (DM) has made great progress in recent years, and its effect has been recognized.^[9]^ According to the findings from recent clinical trials,^[10–12]^ TCM could not only reduce blood sugar levels in patients, but also improve insulin resistance, increase insulin secretion, and regulate glycolipid metabolism.^[13]^ Pharmacological studies reveal that Chinese herbal medicines have been shown to effectively reduce blood glucose and delay the occurrence of complications and improve the quality of life of patients^[14,15]^

Tianqi Jiangtang Capsule is a proprietary Chinese medicine approved by China Food and Drug Administration (CFDA),^[21]^ recommended by Chinese guidelines for the treatment of impaired glucose tolerance.^[16]^ Sun's study showed that after taking Tianqi Jiangtang Capsule for 3 months, the reversal rate of glucose tolerance in Impaired glucose tolerance (IGT) patients was as high as 35.5%. With the prolongation of treatment time, the reversal rate further increased, and by 12 months, the reversal rate reached 62%.^[17]^ Some studies have also shown that in type 2 diabetic patients who have been ineffective with metformin for a long time, after taking Tianqi Jiangtang Capsule, the glycated hemoglobin (HbA1c) of patients decreased by 1.01%.^[25]^ Its medicine consists of Astragalus, Trichosanthin, Ligustrum lucidum, Dendrobium, Ginseng, Earth's bone, Coptis, Hawthorn, Eclipta, and Galla Chinensis. With a large number of clinical trials, animal experiments and modern pharmaceutical research, it could be found that Tianqi Jiangtang Capsule showed an anti-diabetic effect via reducing hyperglycaemia and modifying lipid metabolism as well as less adverse events in the treatment of diabetes.^[18]^ With those data, we can study whether the combination of traditional Chinese and antidiabetic agents - Tianqi Jiangtang Capsule combined with antidiabetic agents hypoglycemic drugs can better lower blood sugar levels and improve patients’ clinical symptoms. Therefore, a sensible systematic review of these trials is of great significance. Although Tianqi Jiangtang Capsule has been studied in the treatment of diabetes mellitus, there is still insufficient evidence. In order to evaluate the safety and effectiveness of Tianqi Jiangtang Capsule in the treatment of type 2 diabetes as well as provide effective evidence for clinical application, this study intends to conduct a systematic review of the clinical randomized controlled trials of Tianqi Jiangtang Capsule in the treatment of T2DM.

## 2 Materials and methods

This systematic review has been registered on PROSPERO as ID148654.

### 2.1 Source of literature

Retrieval of the therapeutic effect of Tianqi Jiangtang Capsule on type 2 diabetes mellitus was conducted in 7 databases including PubMed, Embase, Cochrane Library, China Biomedical Literature CD-ROM Database (CBM), China National Knowledge Infrastructure (CNKI), China Resources Database and Wan Fang. The retrieval time was from the establishment of the database to August 10, 2019.

Two reviewers (SMS and BCR) independently extracted the literature based on the title and abstract of the article. Differences of opinion can be resolved through discussion or consultation with P-CL.

### 2.2 Search method

Two researchers (QZ and DJ) independently conducted literature searches, screened according to inclusion and exclusion criteria, and cross-checked; 2 researchers negotiated to resolve differences, or consult relevant experts to resolve (P-CL) According to different database requirements, the corresponding search formula is adopted. To avoid omission, the search scope includes keyword, keyword or full text. The search terms mainly include (Tianqi Jiangtang Capsule or Tianqi Jiangtang Tablets) and (Diabetes or Diabetes Mellitus or T2DM or Type 2 Diabetes) and (RCT or randomized or randomized controls). Detailed search strategies are listed in the attachment.

### 2.3 Inclusion criteria for study selection

(1) Study object: According to World Health Organization (WHO) and other diagnostic criteria diagnosed as type 2 diabetes;^[19]^
(2) study type: randomized controlled study;
(3) Types of intervention measures.

Control group: Patients in the control group received routine comprehensive treatment or this patients use routine comprehensive treatment combined with other treatments except Tianqi Jiangtang Capsule.

Treatment Group: The treatment group was treated with Tianqi Jiangtang Capsule; (1) Types of outcome indicators (including at least 2 of them): HbA1c, fasting blood glucose (FBG), 2 hours postprandial blood glucose (2hPG), triglyceride (TG), Low density lipoprotein (LDL-C),insulin secretion. The first three items are the main outcome indicators, and the last two are the secondary outcome indicators.

### 2.4 Exclusion criteria for study selection

(1) Repeated publication of the literature;
(2) literature that can be not extract relevant data;
(3) control group also included the treatment of Tianqi Jiangtang Capsule;
(4) animal experiment.

### 2.5 Literature screening and data extraction

The literature screening was performed independently by 2 investigators (LW and CT) based on inclusion and exclusion criteria. The data extraction includes: author, year, test group and control group treatment measures, allocation hidden, whether to use blind method, treatment, whether there are cases of shedding, outcome indicators and adverse reactions. Any discrepancies were resolved by discussion (with P-CL).

### 2.6 Evidence quality evaluation

Literature quality assessments were performed using the Cochrane Risk Bias Evaluation Tool,^[20]^ which was independently assessed by 2 investigators (QZ and DJ). The evaluation criteria include the following6 aspects:

(1) random sequence generation method;
(2) whether allocation concealment is used;
(3) whether the subject and the intervention provider are blinded;
(4) whether the result evaluator is blind;
(5) whether the result data is complete;
(6) Whether selective results reporting and other sources of bias. In case of disagreement, it shall be decided by a third party (with P-CL, L-FM, and T-XL).

The authors used The Grading of Recommendations Assessment, Development and Evaluation (GRADE) methodology to assess the quality of the evidence for each outcome.^[21]^ And each level of evidence will be made “very low,” “low,” “moderate,” or “high” judgment.

### 2.7 Assessment of publication bias

If a result of a meta-analysis contains more than 10 articles and above, we can use RevMan 5.3 software to draw funnel chart and analyze potential publishing deviation with funnel chart.

### 2.8 Data statistics and analysis

Statistical analysis was performed using Review Manager 5.3 software from the Cochrane Collaboration.^[22]^ The measurement data adopts the mean difference (MD), and the categorical variable uses the odds ratio (OR) as the measurement index, with 95% CI as the effect amount. Heterogeneity test was first performed on the included studies. When the heterogeneity test results were *P* > .05 or I^2^ < 50%, the heterogeneity between the studies was not statistically significant. The fixed effect model was used; when the heterogeneity test results *P* ≤ .05 or I^2^ ≥ 50% suggested that the heterogeneity between the studies was statistically significant. The randomized effect model was used to compare the clinical efficacy of T2DM in the experimental group and the control group, and may lead to heterogeneity. The main aspects of the sensitivity analysis were to identify sources of heterogeneity.

## 3 Results

### 3.1 Study characteristics and quality assessment

**Figure 1:**
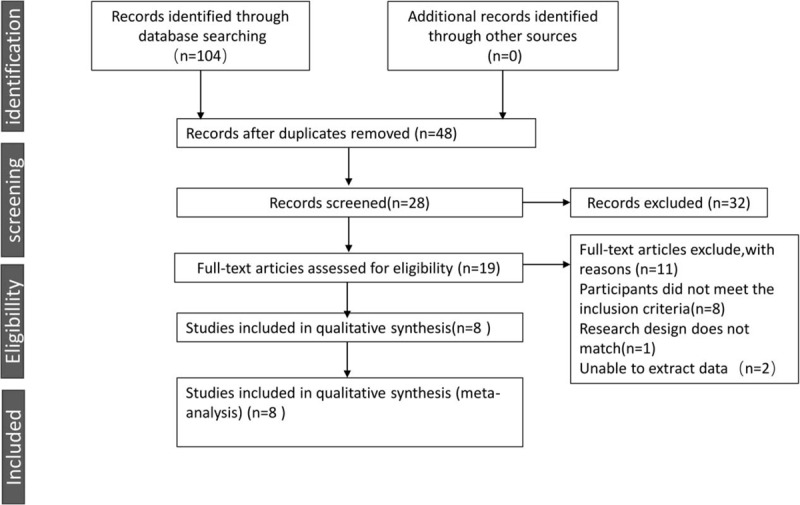
The screening process summarized in a flow diagram.

**Figure 2:**
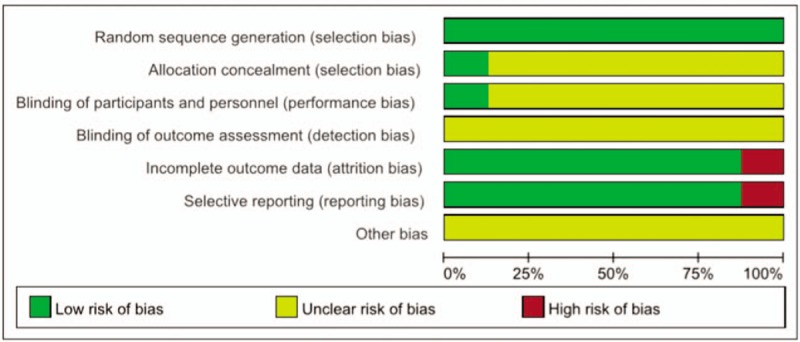
Quality assessment of the included trials-Risk of bias graph.

**Figure 3:**
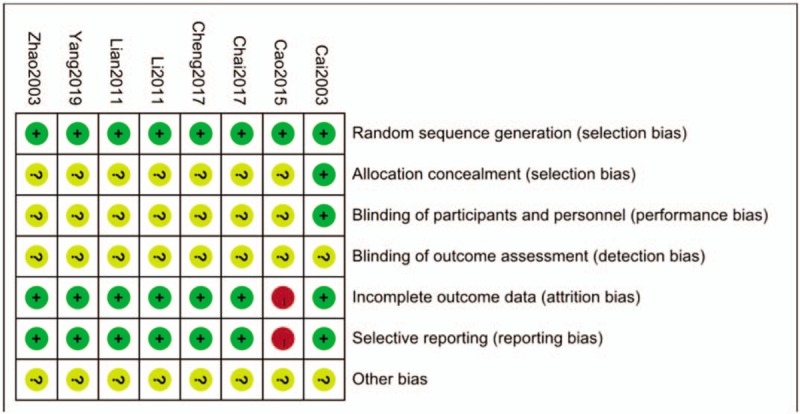
Quality assessment of the included trials-Risk of bias summary.

We have screened 103 pieces of literature through preliminary screening (CNKI = 43, Wanfang = 23, VIP = 20, CBM = 17, PubMed = 1). In these data, 56 were deleted because of duplicate data, 2 were deleted because of unreadable data, and 37 were deleted because the summary did not meet the inclusion criteria.^[23–30]^ The whole process of deletion can be seen in Figure 1. In 8 articles, all the trials were divided into randomized controlled groups. The total number of patients in the treatment group was 606, while that in the control group was 423. Patients were observed for more than 8 weeks. Tianqi Jiangtang Capsule combined with metformin, insulin and gliclazide were compared with placebo or hypoglycemic drugs. (Table 1) HbA1C, FPG, and 2hPG were observed in all 8 trials^[23–30]^ TG and LDL-C was observed in 4 trials,^[23–25,30]^ and Fasting Insulin was observed in 3 trials.^[24,25,27]^Figures 2 and 3 depicts a detailed overview of how each study scored in each category of bias.

**Table 1:**
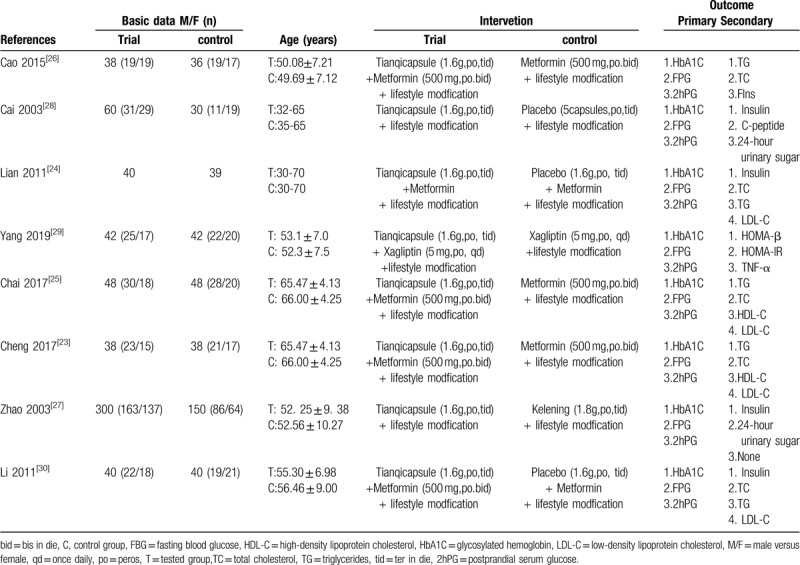
Basic characteristics of included studies.

### 3.2 Primary outcomes

#### 3.2.1 HbA1c

**Figure 4:**
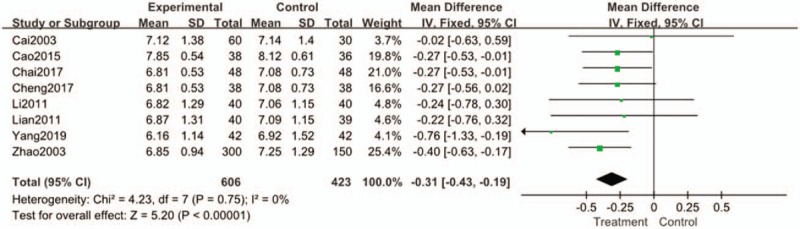
Forest plots of comparison of HAb1c for Tianqi Jiangtang Capsule plus antidiabetic agents therapy versus antidiabetic agents alone.

All the literatures analyzed the HbA1c. The heterogeneity among the experiments was as follows: I^2^ = 0% < 50% (Chi^2^ = 4.23, DF = 7, *P* = .75). It shows that there are significant differences between the treatment group and the control group. (n = 1029; MD, −0.31; 95% CI, [−0.43 to −0.19]; *P* < .00001; I^2^ = 0%) (Fig. 4).

#### 3.2.2 FBG

**Figure 5:**
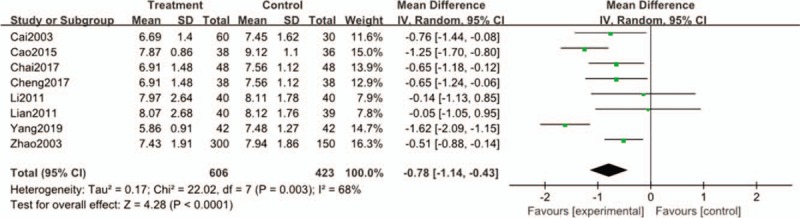
Forest plots of comparison of FBG for Tianqi Jiangtang Capsule plus antidiabetic agents therapy versus antidiabetic agents alone.

All the literatures analyzed FBG. Heterogeneity test results showed that I^2^ = 68% > 50%(Chi^2^ = 22.02, DF = 7, *P* = .003, I^2^ = 68%) had high heterogeneity. Meta-analysis using random effect model showed that total effective (Z = 4.28 (*P* < .0001), MD = 0.78, 95%CI[−1.14 to −0.43]). There was a significant difference between the 2 groups. (Fig. 5).

#### 3.2.3 2hPG

**Figure 6:**
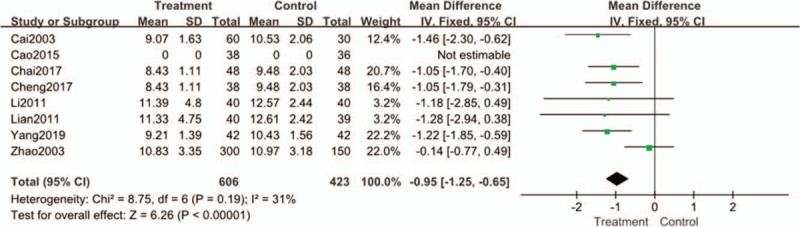
Forest plots of comparison of 2hPG for Tianqi Jiangtang Capsule plus antidiabetic agents therapy versus antidiabetic agents alone.

Heterogeneity test showed that eight literatures were homogeneous [Chi^2^ = 8.75, DF = 8 (*P* = .19); I^2^ = 31%], so fixed effect model was adopted. The results showed that there were significant differences in 2hPG between the treatment group and the control group [OR = −1.25, 95% CI [−1.25 to −0.65], Z = 6.26 (*P* < .00001)]. It indicated that 2hPG in the treatment group taking Tianqi Jiangtang Capsule was significantly higher than that in the control group. (Fig. 6).

### 3.3 Secondary outcomes

#### 3.3.1 Fasting insulin

**Figure 7:**
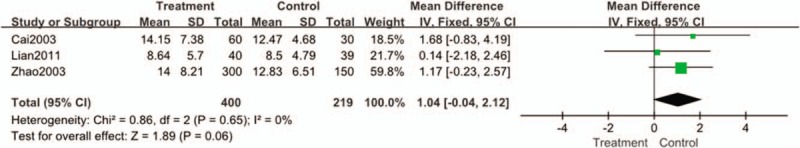
Forest plots of comparison of Fasting Insulin for Tianqi Jiangtang Capsule plus antidiabetic agents therapy versus antidiabetic agents alone.

Fasting Insulin status was recorded in 3 of the 8 articles included. Heterogeneity test showed that the three papers were homogeneous. Thus, a statistical analysis of fixed-effects model was adopted for this outcome. (n = 619; MD, 1.04; 95% CI, [−0.04 to 2.12]; *P* = .651; I^2^ = 0%) However, the number of documents included in this comparison is small, so the evaluation results have certain limitations. (Fig. 7).

#### 3.3.2 TG

**Figure 8:**
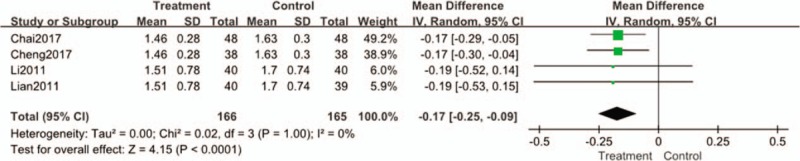
Forest plots of comparison of TG for Tianqi Jiangtang Capsule plus antidiabetic agents therapy versus antidiabetic agents alone.

Four studies reported TG levels. Statistical heterogeneity existed among the results (I^2^ = 0%, *P* = 1). The results showed that the reduced value of TG in the treatment group was inferior to that in the control group. (n = 331; MD, −0.17; 95% CI, [-0.25 to -0.09]; *P* < .00001; I^2^ = 0%) (Fig. 8).

#### 3.3.3 LDL-C

**Figure 9:**
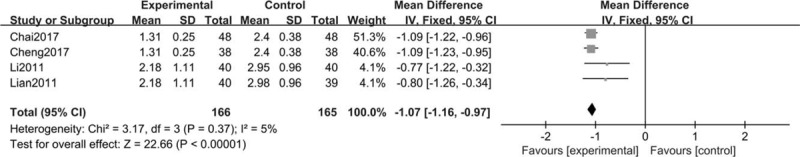
Forest plots of comparison of LDL-C for Tianqi Jiangtang Capsule plus antidiabetic agents therapy versus antidiabetic agents alone.

LDL-C has also been studied in 4 studies. There was no statistical heterogeneity among the studies (I^2^ = 5%, *P* = .37), so the fixed effect model was used. Meta-analysis showed that the therapeutic effect of reducing FPG level in the treatment group was better than that in the control group. (n = 331; MD, −1.07; 95% CI, [−1.16 to −0.97]; *P* < .00001; I^2^ = 5%) (Fig. 9).

### 3.4 Publication bias

**Figure 10:**
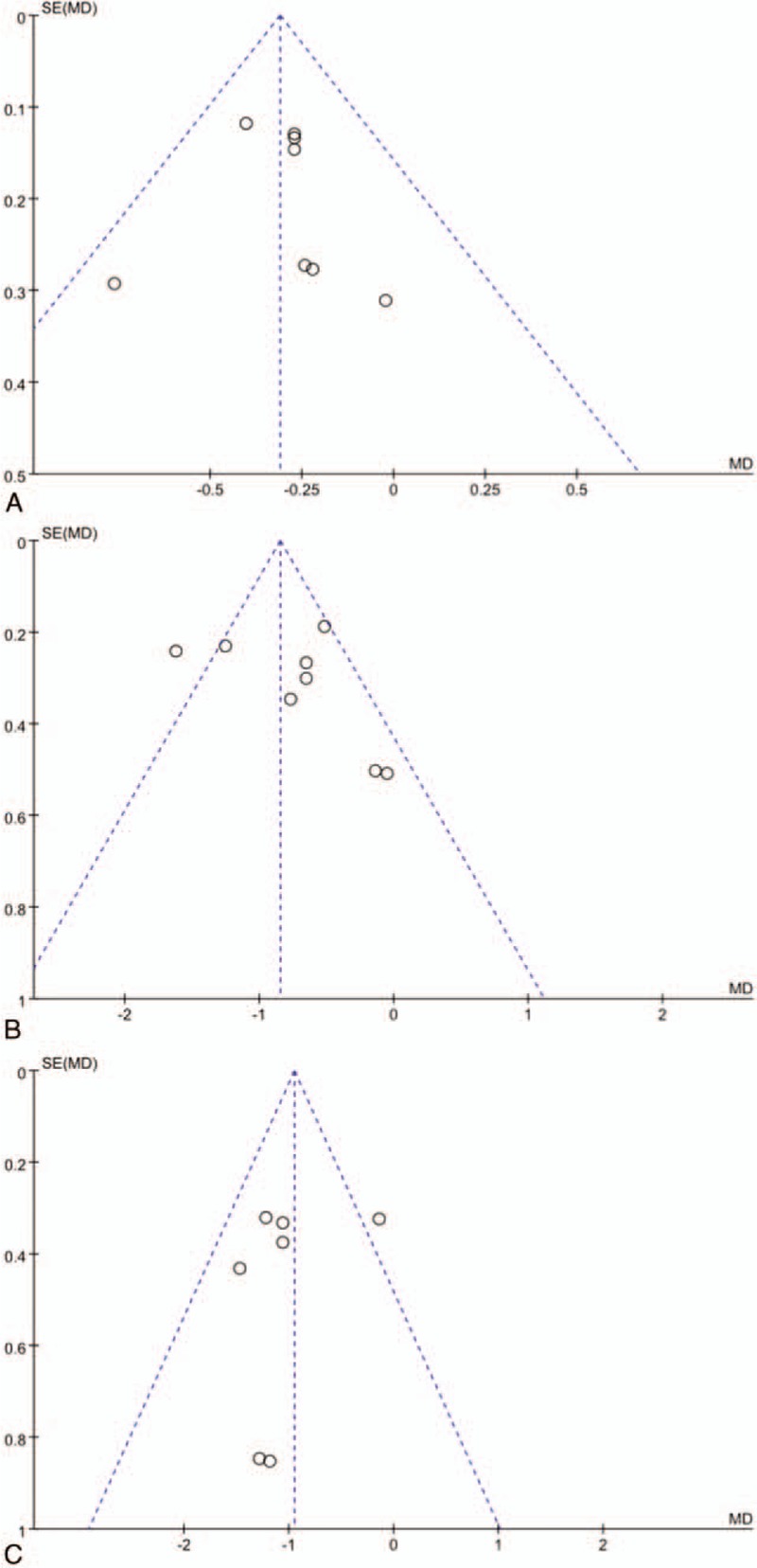
Funnel plot of the trials that compared treatment group with control group; (A) HAb1c; (B) FBG; (C) 2hPG.

HbA1c, FBG, and 2hPG were selected as indicators for the analysis of “inverted funnel diagram”. The distribution of inverted funnel diagrams is within the range of intervals, and the experimental distribution is less symmetrical, suggesting that there may be some publication bias. (Fig. 10A–C).

### 3.5 Evaluation of quality of evidence

**Figure 11:**
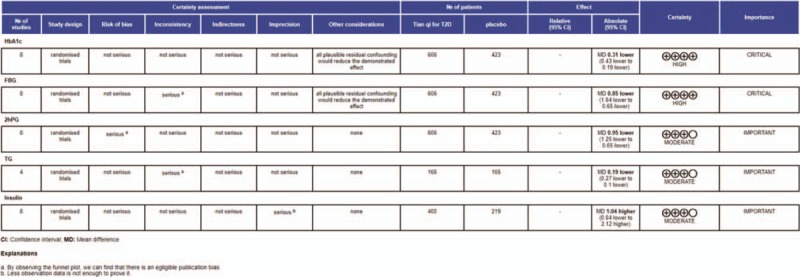
Assessment of quality of evidence.

Figure 11 revealed evidence quality of the included studies of each outcome.^[31]^ The GRADE showed that the evidence quality of Tianqi Jiangtang Capsule and HbA1c, FBG, 2hPG was higher than that of TG and insulin. (Fig. 11).

## 4 Discussion

According to the current American Diabetes Association (ADA) treatment for diabetes, when we treat diabetes, we first choose to improve lifestyle and metformin treatment, and then second-line treatment should choose treatment according to the patient's comorbidities.^[32]^ In the treatment of diabetes, especially in the treatment of its complications, it will directly increase the use of drugs. Whether in life or psychology, it will increase the financial burden of patients. As a special national medicine in China and other Asian countries, TCM also plays a key role in the treatment of diabetes and its complications.^[33]^ This work aims to study the efficacy and safety of traditional Chinese patent medicine-Tianqi Jiangtang Capsule in the treatment of type 2 diabetes, so as to make a systematic evaluation and meta-analysis. In this systematic review, 8 trials involving 1029 participants were included. From the data statistics, we can conclude that Tianqi Jiangtang Capsule combined with basic treatment is more effective in improving patients’ glucose metabolism than basic treatment alone. In addition, the reported adverse events were not serious and no additional special treatment was required.

Studies have shown that every 1% reduction in HbA1c, 12% reduction in stroke risk, 14% reduction in myocardial infarction risk and 12% reduction in diabetes-related mortality risk.^[34]^ The system evaluation takes HbA1c as one of the main analysis indexes. Meta-analysis of HbA1c showed that there were significant differences between the treatment group and the control group. (MD, −0.31; 95% CI, [−0.43 to −0.19]; *P* < .00001; I^2^ = 0%) The meta-analysis results aslo show that the treatment group is better than the control group in reducing the level of HbA1c compared with the control group. The main research shows that Tianqi Jiangtang Capsule combined with antidiabetic agent can provide better benefits for diabetic patients and can significantly reduce HbA1c level compared with antidiabetic agent alone. In addition, animal experiments and clinical trials show that Tianqi Jiangtang Capsule has definite therapeutic effects: enhancing insulin sensitivity, effectively protecting islet β cells; smoothly and effectively reducing blood sugar; comprehensively improving dyslipidemia and preventing complications.^[27,28,40]^

By meta-analysis of FBG in these 8 articles, the results of heterogeneity test showed that there was heterogeneity. It was found that the intervention drug in Yang's article was sitagliptin by combining random effect models.^[11]^ Sitagliptin, one of the DDP-4 inhibitors, affects blood sugar control through a variety of mechanisms, including enhancing glucose-dependent insulin secretion, slowing gastric emptying, and reducing postprandial glucagon secretion and food intake.^[35,36]^ The results may be related to the use of sitagliptin.

Seven studies compared the changes of 2hPG (except Cao's articles), there is a low degree of heterogeneity among the studies (I^2^ = 31%). The results showed that the treatment group was significantly higher than the control group in improving 2hPG. It shows that Tianqi Jiangtang Capsule also has advantages in lowering the level of 2hPG.

Three trials^[24,25,27]^ with 619 participants were included in this work, reporting that the value of Fasting Insulin decreased significantly. And 4 trials^[23–25,30]^ with 331 participants were included in this work, reporting that TG and LDL-C levels also decreased significantly. The most common cause of morbidity and mortality in type 2 diabetes is premature cardiovascular disease (CVD).^[37]^ Statin therapy represents the basis for the management of hypercholesterolemia at present.^[38]^ However, studies have shown that the increased risk of diabetes seems to be associated with high-intensity statin therapy.^[39]^ The potential mechanism of Tianqi Jiangtang Capsule in regulating lipid metabolism was also confirmed by animal experiments. Zhang's experiment showed that after 4 weeks of administration, KKAy mice treated with Tianqi Jiangtang Capsule had more significant improvement in blood lipid than KKAy mice treated with saline (*P* < .05).^[40]^ Therefore, Tianqi Jiangtang Capsule can not only reduce the blood sugar level of patients, but also improve the condition of abnormal blood lipid.

### 4.1 Limitations

Of course, the results of this meta-analysis also have shortcomings. First, we get from the analysis results that FBG results are heterogeneous, which makes the results of analysis and comparison not completely reliable. Second, only 8 RCTs were included in the study through rigorous screening, and all of them were Chinese literatures. This makes the results not comprehensive enough and may lead to deviations in the results. Third, although all the patients in the study were type 2 diabetes mellitus, there were many kinds of intervention drugs and different intervening time, which would lead to deviations in the results. Therefore, the findings might have limitations for subjects outside China and should be interpreted with caution.

## 5 Conclusion

Systematic review shows that the curative effect of Tianqi Jiangtang Capsule combined with antidiabetic drugs is greater than that of antidiabetic drugs alone, especially on HbA1c and blood lipid. However, the evidence of quantity and quality of research is still insufficient. More rigorous clinical trials are needed to illustrate the advantages and benefits of Tianqi Jiangtang Capsule in the treatment of type 2 diabetes mellitus.

## Author contributions

**Data curation:** Chunli Piao, Qi Zhang, De Jin.

**Formal analysis:** Mengsu Shao, Chaoran Bi, Li Wang, Cheng Tang.

**Project administration:** Chunli Piao, Fengmei Lian, Xiaolin Tong.

**Supervision:** Chunli Piao, Fengmei Lian, Xiaolin Tong.

**Writing – original draft:** Chunli Piao, Qi Zhang, De Jin.
